# Utilization of the flow ratio measured by echocardiography (FR_echo_) compared to the flow ratio by right heart catheterization (FR_rhc_) for detecting Eisenmenger syndrome in uncorrected acyanotic adult congenital heart disease (ACHD)

**DOI:** 10.1186/s12880-025-01651-y

**Published:** 2025-04-25

**Authors:** Charlotte Johanna Cool, Achmad Fitrah Khalid, Norman Sukmadi

**Affiliations:** Department of Cardiology and Vascular Medicine, Faculty of Medicine, University of Padjadjaran, Jalan Pasteur No 38, Pasteur, Bandung, Jawa Barat Indonesia

**Keywords:** Uncorrected adult congenital heart disease, Flow ratio, Transthoracic echocardiography, Right heart catheterization surrogate

## Abstract

**Background:**

The increasing number of adult congenital heart disease (ACHD) patients, especially in low- and middle-income countries (LMICs), necessitates effective management methods. The Qp/Qs or flow ratio (FR) is crucial for this purpose because one of the indications for closure is a significant shunt. This study compares the utility of the transthoracic echocardiography (TTE)-derived flow ratio (FR_echo_) with that of the right heart catheterization (RHC)-derived flow ratio (FR_rhc_) to guide clinical decisions in resource-limited settings.

**Materials and methods:**

This cross-sectional study in Bandung, Indonesia, included 36 patients with uncorrected acyanotic ACHD who underwent both RHC and TTE on the same day. FR_echo_ was calculated using stroke volumes of the respective ventricles derived from Doppler measurements, and FR_rhc_ was measured using indirect Fick’s method.

**Results:**

Of the 36 patients, 80.6% were female, with a median age of 31 (18–65) years. The majority had secundum atrial septal defects (61.1%). The mean FR_echo_ was 2.8 ± 1.5 and the median FR_rhc_ was 1.69 (0.46–3.89). FR_echo_ showed a significant positive correlation with FR_rhc_ (ρ = 0.656, *p* < 0.001). Bland‒Altman analysis revealed a mean difference of 1 (-1.4–3.3). Subgroup analysis of patients with a FRrhc shunt < 1 showed a mean difference of 0.7 (-1–2.3).

**Conclusion:**

TTE-derived FR_echo_ tends to overestimate FR compared to FR_rhc_. FR_echo_ should not be used as a surrogate for FR_rhc_ in this population.

## Background

The number of patients with adult congenital heart disease (ACHD) is increasing due to advances in diagnostic and therapeutic modalities. Uncorrected congenital heart disease is more common in low- and middle-income countries (LMICs) due to late diagnosis, economic problems, or a lack of education about the importance of defect correction [1].

Acyanotic ACHD represents a spectrum of clinical presentations, ranging from asymptomatic pulmonary arterial hypertension (PAH) to Eisenmenger syndrome (ES), characterized by reversal (pulmonary-to-systemic) or bidirectional shunting due to severe elevation of pulmonary vascular resistance (PVR). The Qp/Qs ratio, or flow ratio (FR), is a tool for quantifying shunt direction and significance. A FR < 1 indicates a dominant right-to-left shunt, while a FR > 1 indicates a dominant left-to-right shunt [2, 3].

Providing optimal care for ACHD patients is challenging, especially in resource-limited settings such as Indonesia [4]. Diagnostic and therapeutic interventions often require a high level of expertise and resources. According to the 2020 ESC Guidelines for the Management of Adult Congenital Heart Disease, the measurement of PVR, and FR measurement is an integral part of the indication for defect closure [5]. Magnetic resonance imaging (MRI) is a good alternative for noninvasively measuring FR. However, radiation exposure, high cost, and machine availability are limitations in developing countries. On the other hand, transthoracic echocardiography (TTE) is a modality that is easy to find and widely used in diagnosing CHD patients and finding other necessary information [6]. Measuring the pressure gradient between ventricles or great arteries could be used to determine the direction of the shunt, especially in VSD or PDA, since the pressure gradient between the defects is high. However, these methods are not applicable in ASD patients, and clinicians often rely on color Doppler to qualitatively determine the direction of the shunt since the pressure gradient between the atria is low [7].

FR can be measured by TTE as the cross-sectional area times the average velocity of the blood passing through the vessel from the right ventricle and the left ventricle [8]. TTE estimation of FR could be used to estimate shunt flow, especially in ASD patients. However, data regarding the measurement of FR by TTE compared to RHC is scarce. In this study, we aimed to explore the utility of TTE in assessing FR compared to RHC for guiding cardiologists in LMICs, especially in low-resource settings, in making clinical decisions.

## Materials and methods

This cross-sectional study was conducted in Bandung, Indonesia, in accordance with the Declaration of Helsinki and was approved by the Hasan Sadikin Hospital Ethics Committee, and written informed consent was obtained from each participant. Consecutive patients with uncorrected acyanotic ACHD who underwent RHC procedures and echocardiography on the same day at Hasan Sadikin Hospital Bandung from 2023 to 2024 composed the population. The inclusion criterion in this study was adults ≥ 18 years. We excluded those with complex congenital heart disease, great arteries level shunt, poor echo window, pregnancy, or an LVEF < 50% on echocardiography.

### Echocardiography

A Philips EPIQ CVx was used. The flow quantification (Q) by TTE was derived from stroke volume (SV) multiplied by heart rate (HR). Qp is the pulmonic flow, derived from the pulmonic (right ventricle) SV multiplied by the HR, and Qs is the systemic flow, derived from the systemic (left ventricle) SV multiplied by the HR. Furthermore, the equation was simplified as Q = SV of the respective ventricle since HR was consistent between ventricles. FR or Qp/Qs measured by echo (FR_echo_) was the ratio of RVSV/LVSV.

SV was calculated as the cross-sectional area (CSA) multiplied by the velocity time integral (VTI) of the respective ventricle. RVOT VTI was obtained by placing a pulse wave (PW) Doppler sample within the pulmonic valve from the parasternal short-axis view. The RVOT diameter was obtained at the end diastolic phase, measured at its distal using the inner edge-to-inner edge technique in 2D measurement at the PSAX view. LVOT VTI was obtained by placing the PW Doppler device within the aortic valve from the apical 5-chamber view. LVOT diameter was measured at the same location at which the highest velocity signal was obtained. All the echocardiographic measure were measured in concordance with American Society of Echocardiography (ASE) guidelines [8]. All the acquired data were validated by a certified congenital heart disease cardiologist.

### Right heart catheterization

Right heart catheterization was performed using femoral vein access. Continuous measurements of pressure and oxygen saturation were performed. FR was calculated using indirect Fick’s method as Qp/Qs and defined as Eisenmenger when the FR < 1. All examinations were performed by a congenital heart disease specialist as a single observer.

### Statistical analysis

The analysis started with descriptive analysis and the normality test of numerical variables in the form of the Kolmogorov‒Smirnov test. Normally distributed data are expressed as the mean ± standard deviation (SD); nonnormally distributed data are expressed as the median (range min – max). Paired t tests or Mann‒Whitney tests were used to determine differences between the FRrhc groups, with a cutoff of 1. Pearson correlation tests or Spearman’s rho tests were used depending on the normality of the data to determine the correlation between FR_echo_ and FR_rhc_. Bland‒Altman analysis was used to assess bias, with clinically acceptable results of -0.5 and 1.5. If there was good agreement, we analyzed its specificity, sensitivity, negative predictive value, and positive predictive value with a simple 2 × 2 table. The data obtained were recorded in a special form and then processed through the IBM SPSS version 25.0 program for 64-bit Windows.

## Results

A total of 36 patients were included in the study; 29 (80.6%) participants were female, with a median age of 31 (18–65) years. Most patients were diagnosed with Secundum ASD (61.1%). The median room air oxygen saturation was 94 (71–99) %. The median RVOT VTI was 16.6 (7–46.2) cm, and the mean RVOT diameter was 30.4 ± 5.7 mm. The mean RV basal height was 50 (38–77) mm. The mean pulmonary artery pressure measured by RHC was 57.5 ± 22.2 mmHg. The median PVR according to the RHC was 10.5 (0.13–33.9) WU. For detailed information, see Table 1.

**Table 1:** Baseline characteristics

Characteristics	*N* = 36
Age, median	31 (18–65)
Sex (female), n (%)	29 (80.6)
Body Mass Index (kg/m^2^), median	19 (14.7–30.2)
Oxygen Saturation (%), median	94 (71–99)
Diagnosis, n (%)	
Primum ASD	3 (8.3)
Secundum ASD	22 (61.1)
AVSD	1 (2.8)
Inlet VSD	2 (5.6)
PM VSD	6 (16.7)
SADC VSD	2 (5.6)
Shunt, n(%)	
Left to Right Shunt	18 (50)
Bidirectional Shunt	14 (38.7)
Balanced Shunt	4 (11.3)
RVOT VTI (cm), median	16.6 (7–46.2)
RVOT diameter (mm), mean ± SD	30.4 ± 5.7
Probability of PH, n(%)	
Low	2 (5.6)
High	34 (94.4)
RV Basal (mm), median	50 (38–77)
TR Vmax (m/s), mean ± SD	4 ± 0.9
PVAccT, median	80 (34–178)
FRecho, mean ± SD	2.8 ± 1.5
mPAP (RHC) (mmHg), mean ± SD	57.5 ± 22.2
PVR (RHC) (WU), median	10.5 (0.13–33.9)
FRrhc, median	1.69 (0.46–3.89)
PVR/SVR ratio (RHC), median	0.338 (0.01–1.75)

ASD: Atrial septal defect, AVSD: Atrio-ventricular septal defect, FR: Flow ratio, mPAP: Mean pulmonary artery pressure, PH: Pulmonary hypertension, PM: Perimembranous, PVAccT: Pulmonic valve acceleration time, RHC: Right heart catheterization, RV: Right ventricle, RVOT: Right ventricle outflow tract, SADC: Sub-arterial doubly committed, SVR: Systemic vascular resistance, TR V Max: Tricuspid regurgitation maximal velocity, VSD: Ventricular septal defect, WU: Wood units

Right heart catheterization was conducted and found a significant difference in all RHC parameters between ES and non-ES. Table 2. Furthermore, patients were grouped based on correctability according to current ESC ACHD guidelines. No significant differences were found in age, BMI, RV basal, TR Vmax, PVAccT, and FRecho between the groups (*p* = 0.203, *p* = 0.077, *p* = 0.095, *p* = 0.05, *p* = 0.071, *p* = 0.105, respectively). The median peripheral oxygen saturation was significantly lower in non-correctable group [92 (71–99) vs. 96 (92–99), *p* < 0.001]. FR_echo_ was significantly lower in non-correctable group [1.3 (0.46–3.89) vs. 2.97(1.5–3.75), *p* = 0.002] Table 3.

**Table 2:** Differences in RHC parameters between groups

Parameters	Eisenmenger (*n* = 8)	Non-Eisenmenger (*n* = 28)	*P* Value
Systolic PAP	114.6 ± 15.2	88.7 ± 31.5	0.003
mPAP	74.9 ± 15.5	52.5 ± 21.4	0.010
FR*	0.62 (0.46–0.9)	2 (1.05–3.89)	< 0.001
PVR*	25.2 (16.2–33.9)	7.1 (0.13–20.97)	< 0.001
SVR	21.8 ± 4	29.7 ± 8.2	0.013
CO	4.1 ± 1	3.2 ± 0.9	0.009
PVR/SVR*	1.17 (0.81–1.75)	0.25 (0.01–0.71)	< 0.001

*Mann‒Whitney test was used

CO: cardiac output, FR: flow ratio, mPAP: mean pulmonary artery pressure, PAP: pulmonary artery pressure, PVR: pulmonary vascular resistance, SVR: systemic vascular resistance

**Table 3:** Differences in parameters between correctability

Parameters	Correctable (*n* = 11)	Non-Correctable (*n* = 25)	*P* Value
Age*	39 (20–58)	29 (18–65)	0.203
BMI*	20.9 (15.1–30.2)	18.5 (14.7–25.4)	0.077
SpO2*	96 (92–99)	92 (71–99)	0.002
RV basal*	46 (38–58)	53 (40–77)	0.095
TR V max	3.5 ± 0.8	4.2 ± 0.8	0.05
PVAccT*	95 (71–178)	79 (34–155)	0.071
FRecho	3.4 ± 1.5	2.5 ± 1.5	0.105
FRrhc*	2.7 (1.5–3.75)	1.3 (0.46–3.89)	0.011

*Mann‒Whitney test was used

BMI: body mass index, FR: flow ratio, PVAccT: pulmonic valve acceleration time, RHC: right heart catheterization, RV: right ventricle, SpO2: Peripheral oxygen saturation, TR V Max: Tricuspid regurgitation maximal velocity

**Fig. 1:**
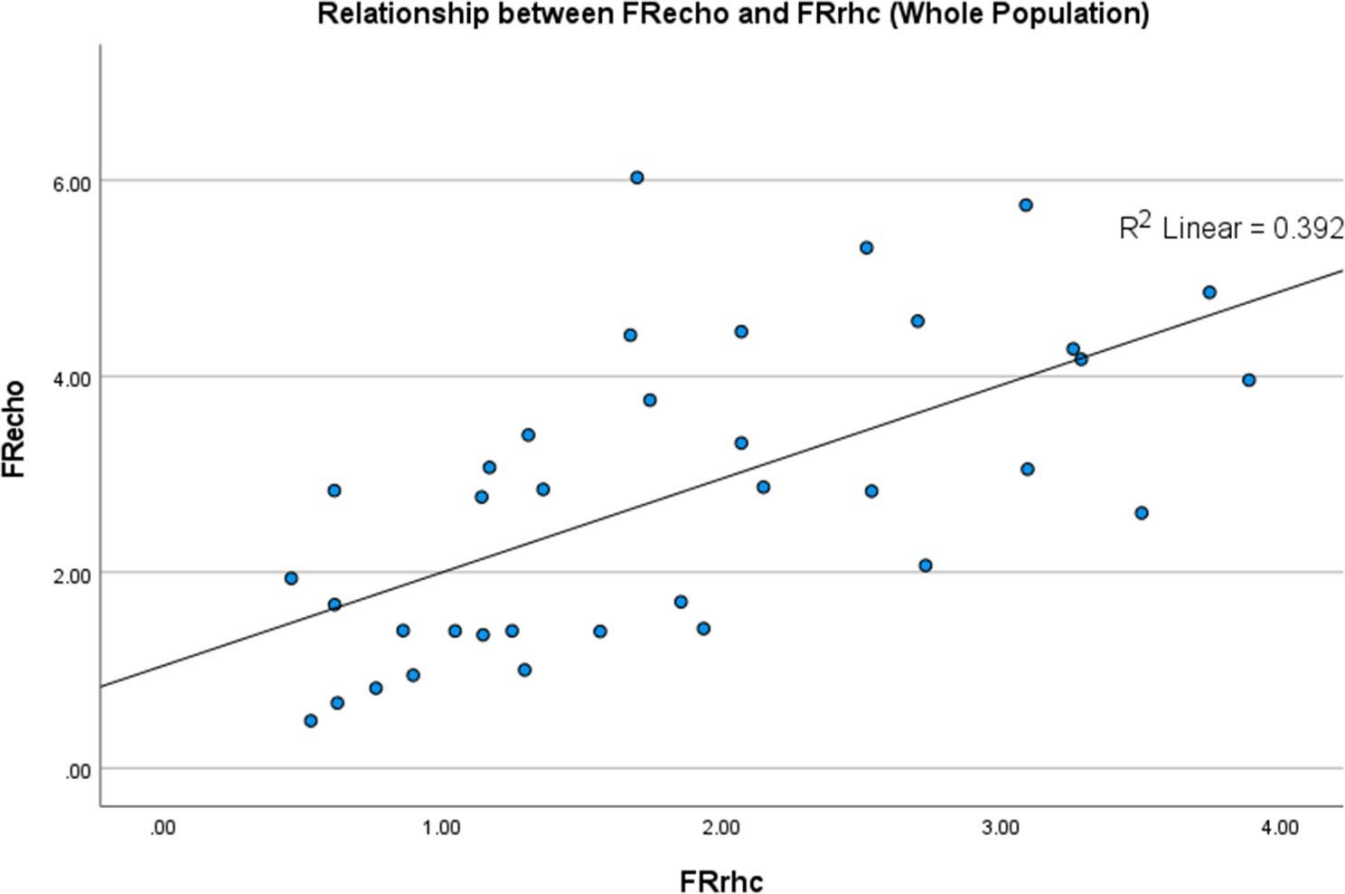
Relationship between FRecho and FRrhc

**Fig. 2:**
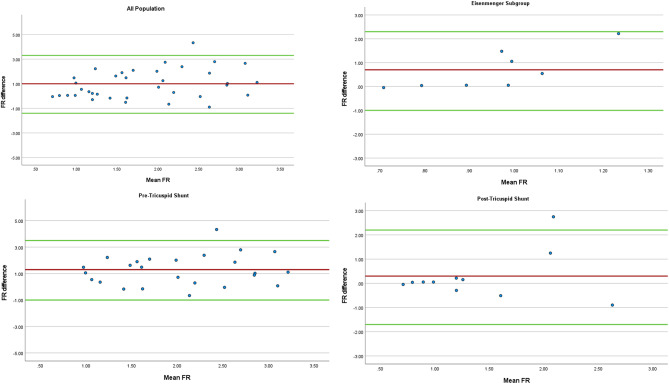
Bland‒Altmann analysis between FRecho and FRrhc

Spearman’s rho bivariate analysis was performed and revealed a significant positive correlation between FR_echo_ and FR_rhc_ (*p* < 0.001, ρ = 0.656). Moreover, we make subgroup bivariate analysis based on the pre-tricuspid shunt and post tricuspid shunt. We found significant positive correlation between the FR_echo_ and FR_rhc_ from pre-tricuspid shunt and post tricuspid shunt subgroup (*p* < 0.001, ρ = 0.594 and *p* < 0.001, ρ = 0.909, respectively) Fig. 1. Furthermore, we used Bland‒Altman analysis to assess bias. The overall mean difference in the whole population was 1 (-1.4–3.3), indicating good agreement but not meeting the a priori clinically accepted results. Subgroup analysis of patients with a FR_rhc_ shunt < 1 was performed. The results were similar, with a mean difference of 0.7 (-1–2.3). Additionally, we performed subgroup analysis of patients with pre- and post- tricuspid shunts. The results were not clinically accepted, with mean difference of 1.3 (-1–3.5) and 0.3 (-1.7–2.2) respectively Fig. 2.

## Discussion

Accurate measurement of FR is crucial for the management of uncorrected acyanotic adult congenital heart disease. The European Society of Cardiology (ESC) recommends the FR threshold for closure in patients with ASD, VSD, and PDA. Patients who experienced a FR < 1.5 were considered to be at risk of defect closure [6]. Color flow Doppler mapping is usually performed to determine the direction of flow in daily practice. However, this method lacks precision compared to quantitative measurements, for instance, FR calculated using the ratio of right ventricular stroke volume (RVSV) to left ventricular stroke volume (LVSV) [9]. 

Theoretically, flow (Q) measured by echocardiography is derived from the CSA and the average blood cell velocity through the valve orifice using PW Doppler during the flow period. This measurement is more accurate under a laminar flow with a flat profile. VTI was traced from the outer edge of the brightest portion and averaged over several cardiac cycles, especially in patients with atrial fibrillation [10]. 

In our study, we found that FR_echo_ was poorly correlated clinically with FR_rhc_ and tended to overestimate the value of FR, despite a good positive correlation between the tests. Therefore, FR_echo_ should not be used to estimate FR in this population. This observation was discordant with some previous findings that the Doppler index of the RVSV/LVSV was clinically useful for estimating the FR, especially in ASD patients [11, 12]. 

This discrepancy could be attributed to significant regurgitation in the aortic or pulmonic valve [12]. Furthermore, the different positions of the PW Doppler sample can significantly affect the measurements. High cardiac output can also alter RVOT VTI measurements [5]. 

The last possible explanation for this result is the accuracy of anatomical measurements, especially the RVOT diameter. The American Society of Echocardiography (ASE) reported that the pulmonary annulus is the most difficult area to assess because of poor visualization of the annulus diameter and because the RVOT contracts during systole. In contrast with the RVOT, the LVOT has little variability during systole and therefore has a more precise measurement [10]. 

Our findings were similar to those of Faherty et al., who reported that FR_echo_ has poor correlation and agreement and tends to overestimate the degree of shunting. In their study, a pediatric population was included, and the time interval between TTE and RHC ranged from 58 days. They hypothesized that the results were due to errors in measuring the anatomy [13]. Similarly, we also hypothesized that the overestimation of FR was due to the complex anatomy of the RVOT and limitations of the acoustic window for adequate quantification of the echo [14]. Estimation of cross-sectional areas in the RVOT was more difficult in the presence of the dilated main pulmonary artery, which is commonly present in pulmonary arterial hypertension [15]. 

Our study has several limitations. First, we did not perform an interrater reliability test, which might introduce observer bias, especially in RVOT measurements. Our study was also conducted in a single center; therefore, the results may not be generalizable.

## Conclusion

Although it is theoretically feasible to measure FR by echocardiography, the results were not clinically acceptable in uncorrected acyanotic ACHD patients. FR_echo_ using the RVSV/LVSV tends to overestimate the FR value and cannot be used as a surrogate parameter of the FR_rhc_ despite its availability.

## Data Availability

The datasets used and/or analyzed during the current study are available from the corresponding author upon reasonable request.

## Abbreviations

ACHD: Adult Congenital Heart Disease
ASD: Atrial Septal Defect
AVSD: Atrioventricular Septal Defect
BMI: Body Mass Index
ESC: European Society of Cardiology
FR: Flow Ratio
FR_echo_: Echocardiography-Derived Flow Ratio
FR_rhc_: Right Heart Catheterization-Derived Flow Ratio
LMICs: Low- and Middle-Income Countries
LVOT: Left Ventricular Outflow Tract
mPAP: Mean Pulmonary Artery Pressure
PAH: Pulmonary Arterial Hypertension
PDA: Patent Ductus Arteriosus
PH: Pulmonary Hypertension
PVR: Pulmonary Vascular Resistance
PVAccT: Pulmonic Valve Acceleration Time
Qp/Qs: Pulmonary to Systemic Flow Ratio
RHC: Right Heart Catheterization
RV: Right Ventricle
RVOT: Right Ventricular Outflow Tract
SV: Stroke Volume
SVR: Systemic Vascular Resistance
TTE: Transthoracic Echocardiography
TR Vmax: Tricuspid Regurgitation Maximal Velocity
VSD: Ventricular Septal Defect
VTI: Velocity Time Integral
WU: Wood Units
